# Strategies To Modulate Heritable Epigenetic Defects in Cellular Machinery: Lessons from Nature

**DOI:** 10.3390/ph6010001

**Published:** 2012-12-27

**Authors:** Ganesh N. Pandian, Hiroshi Sugiyama

**Affiliations:** 1 Institute for Integrated Cell-Material Sciences (WPI-iCeMS), Kyoto University, Sakyo, Kyoto 606-8502, Japan; E-Mail: ganesh@kuchem.kyoto-u.ac.jp; 2 Department of Chemistry, Graduate School of Science, Kyoto University, Sakyo, Kyoto 606-8501, Japan; E-Mail: hs@kuchem.kyoto-u.ac.jp

**Keywords:** epigenetic inheritance, chemical mimics, HDAC inhibitors, regenerative medicine, cancer treatment, programmable genetic switches, histone code, epigenetic switch based therapy, future medicine.

## Abstract

Natural epigenetic processes precisely orchestrate the intricate gene network by expressing and suppressing genes at the right place and time, thereby playing an essential role in maintaining the cellular homeostasis. Environment-mediated alteration of this natural epigenomic pattern causes abnormal cell behavior and shifts the cell from the normal to a diseased state, leading to certain cancers and neurodegenerative disorders. Unlike heritable diseases that are caused by the irreversible mutations in DNA, epigenetic errors can be reversed. Inheritance of epigenetic memory is also a major concern in the clinical translation of the Nobel Prize-winning discovery of induced pluripotent stem cell technology. Consequently, there is an increasing interest in the development of novel epigenetic switch-based therapeutic strategies that could potentially restore the heritable changes in epigenetically inherited disorders. Here we give a comprehensive overview of epigenetic inheritance and suggest the prospects of therapeutic gene modulation using epigenetic-based drugs, in particular histone deacetylase inhibitors. This review suggests that there is a need to develop therapeutic strategies that effectively mimic the natural environment and include the ways to modulate the gene expression at both the genetic and epigenetic levels. The development of tailor-made small molecules that could epigenetically alter DNA in a sequence-specific manner is a promising approach for restoring defects in an altered epigenome and may offer a sustainable solution to some unresolved clinical issues.

## 1. Introduction

Life is estimated to have appeared on Earth approximately 3.8 billion years ago [1]. Life has evolved since then through a wide range of decisive patterns and systems of interrelated elements [2,3]. Living systems are incredibly dynamic and are evolving constantly to meet the demands needed to sustain them. A closer look at Nature and a better understanding of how systems maintain themselves could reveal some of the systematic patterns and recurring processes that sustains life and living cells [4]. Thus, Nature could inspire us to mimic some of its decisive patterns that control complex systems. These cues taken from Nature could help us develop sustainable strategies to solve some of the unresolved issues that challenge humankind. One such remarkable natural phenomenon is the precise capability of living cells to develop and differentiate into distinguishable cell types despite containing identical information in their DNA [5]. Considering the limited capability of mammalian systems to regenerate a lost body part or organ, comprehensive understanding of the mechanisms that characterize these phenotypic and functional cellular differences could lead to sustainable therapeutic approaches to certain diseases [6,7].

It is generally accepted that these dynamic cellular differences are controlled by the intricate gene regulatory system [8]. Several studies in the past have suggested that the genetic code alone is insufficient to govern differential gene expression pertaining to cell fate; this notion has been substantiated with the completion of the Human Genome Project [9]. A more complex phenomenon that includes a secondary layer of cellular material surrounding the DNA, termed the epigenome is now accepted to operate a mechanism that controls cell fate by modulating the transcriptional “ON-OFF” status [10]. Recent reports have implied an essential role of the epigenome in dictating differential gene expression [11]. During development, these epigenetic modifications act as a switch to send precise signals to the cells about the critical decision to enter a proliferation state or a differentiation state [12].

Environmental factors such as diet, stress and pre-natal nutrition are known to induce heritable adaptations in the epigenome. Such external factors could make an imprint on genes that could be passed from one generation to the next [13]. These lifestyle-associated aberrant epigenetic adaptations could disrupt cellular machinery and cause profound alterations in the genome-wide gene expression, which may shift the cell from a normal to a diseased state [14].

Causal mutations that occur in DNA are generally difficult to repair. The technique of correcting the defected genes in the body by replacing them with the insertion of new genes, termed gene therapy, offers treatment options for diseases such as cancer and hepatitis. However, immune rejection of vectors and rapid division of cells is a major concern. In addition, some diseases are not caused by a mutation of a single gene. Hence, gene therapy is not effective against multigene disorders like heart disease, diabetes, high blood pressure, Alzheimer`s disease and arthritis [15]. By contrast, changes in the epigenome can be reversed. Epigenetic regulation includes two major types of modifications: 1) methylation in DNA, which is generally stable, and 2) methylation and acetylation of histones, which are relatively flexible [16].

Neurogenerative disorders and certain cancers are major diseases that are associated with epigenetic dysregulation [17]. Because epigenetic modifications can influence gene expression at various distinctive levels, epigenetic-based drugs might be used therapeutically to alleviate certain ailments [18]. Accordingly, employment of epigenetic-based drugs as a therapeutic strategy to cure such etiological diseases has blossomed in recent years [19]. Here, we review the progress in and prospects of epigenetic switch-based strategies for restoring defects in the cellular machinery. Since epigenetic-based drugs alone may be insufficient for effective treatment, this review suggests the need to gain inspiration from nature to design epigenetic drugs that are complemented with selective DNA recognition ability at various distinct levels. We propose that the development of precisely tunable small molecules possessing both the ability for selective gene recognition and for inducing site-specific epigenetic modifications may serve as a sustainable solution for the treatment of epigenetically inherited disorders.

## 2. Epigenetic Inheritance and Heritable Disorders

### 2.1. A Brief History of Epigenetic Inheritance and Their Influence on Cellular Homeostasis in Animal Models

In 1942, C.H. Waddington, who was then working at the interface of developmental biology and genetics, first coined the term “epigenetics” to characterize the programmed differentiation of embryonic cells into functionally distinct cell types [20]. The definition of epigenetics has evolved over time, and epigenetics now refers to transmissible changes in gene expression or cellular phenotype beyond the basic structure of DNA. Epigenetic inheritance is a component of epigenetics, in which the heritable changes induced in the parents by environmental cues induce variations in their offspring without altering the primary structure of DNA and the persistence of those inducing signals even in the present environment [21]. Although epigenetic inheritance has gained importance only in the past few years, the notion that the environment can influence heritable changes in living cells within a generation or two was proposed in as early as the 18^th^ century by naturalist Jean-Baptiste Lamarck. Some notable Lamarckian examples include the inheritance of gradually acquired traits such as the development of long necks in giraffes when they had to reach high leaves and the inheritance of muscles in a blacksmith`s son [22]. Later in the 19^th^ century, psychologist and physician Ivan Pavlov substantiated the Lamarckian principles with a remarkable discovery that when a mouse had learned to navigate a maze, its children and grandchildren inherited the acquired external memory and learnt the maze faster than their parents [23]. In 1956, Huxley noted the patterned dynamic code that may have caused the variation in specification of cellular phenotype, which was not gene centric [24]. In 1970, the ability of different DNA methylation pattern to alter the phenotypic features of a living system was first demonstrated [25]. In 2003, Waterland *et al*. showed the prominent effect on DNA methylation by the dietary supplement containing B vitamins, which has the ability to act as the methyl donors. Interestingly, DNA methylation caused by the B vitamins facilitated the generation of healthy pups, which were not prone to diabetes [26]. These evidences suggested that the epigenetic marks acquired by an individual through the interaction with its environment might be accumulated in a transmissible code [27].

Feig and colleagues showed that the information stored in the epigenome might provide an adaptive advantage to the offspring. By inducing neurotransmission in the mice that had problems with genetic memory, they have shown that even memory could be influenced by external stimuli [28]. Insects provide an ideal model system to rapidly evaluate drugs and dietary supplements as insects could manifest easily distinguishable morphological changes. In a study to evaluate the effect of a drug across generations, the antibiotic geldanamycin was fed to fruit flies, whose eyes showed heritable changes with out alteration in their genome up to until 13 generations [29]. Jablonka and colleagues have shown that visible phenotypic differences could be observed in roundworms fed with a bacteria diet and, interestingly, this epigenetic inheritance endured for at least 40 generations [30]. Self-sustaining regulation of gene products can induce patterned transcriptional activity, which can be transmitted during cell division and were shown to affect phenotype of fungi, microbes and the development of multicellular organisms [13]. Interaction of small RNAs with chromatin was also shown to induce chromatin modifications that could be maintained and were heritable across generations [31]. Among the above-mentioned heritable chromatin modifications, epigenetic inheritance of chromatin marks including histone modification is therapeutically appealing as they can tinker directly with the DNA and introduce their marks in the daughter cells [32].

### 2.2. Nature versus Nurture

For the past few decades, compelling evidence from several studies has indicated that nurture can restore transgenerational epigenetic errors. Behavioral and visual deficits induced by malnutrition could be reversed partly by switching the diet of rodents from an inadequate to normal diet [33]. In accordance with this phenomenon, recently, Carone *et al*. used genome-wide gene analysis to show that the epigenetic landscape of sperm cells of male mice fed with low protein/high sugar diet could be reset through transgenerational environmental reprogramming [34].

Maternal care also plays a fundamental role in the behavior of mammals as a newborn first becomes acclimatized to the environment through its interactions with its mother. In rats, mothers show care by licking and grooming (LG) the pups. A study was carried out to evaluate the effect of varying the level of LG (high, mid and low) on the behavior of the pups. The offspring raised by the low-LG mothers showed elevated corticotrophin-releasing hormone, suggesting an increased response to stress in both the pup and adult stages [35]. By contrast, offspring of the high-LG mothers showed elevated expression of NGFI-A, which is responsible for altered methylation and acetylation patterns in the promoter region of the glucocorticoid receptor. These epigenetic alterations were suggested to shift the signalling cascades to induce different behavioral responses to stress. Interestingly, allowing the high-LG mothers to take over and rear the low-LG pups restored the epigenetic alteration and the response to stress [36]. The above-mentioned results indicate that nurture alone can shift the epigenetic alterations and restore the deficits in cellular transcriptional machinery at least in the animal models.

### 2.3. Effect of Environmental Cues on Transgenerational Epigenetic Damage in Humans

The actual existence of the transgenerational epigenetic damage in humans is yet to be validated. However there are examples of transgenerational inheritance of epigenetic marks in humans [13]. Some of the environmental cues that could influence heritable changes in histone modifications include smoking, diet, stress and social environment. Lifestyle plays a prominent role in maintaining the health status of a living system. Overexposure to cigarette smoke can cause tissue injury through DNA damage, which in turn leads to epigenetic alterations; these aberrant changes in the epigenome progressively shift lung cells from the normal to a diseased state [37]. In 2006, Pembrey and colleagues reported the effect of early smoking on puberty and the ability of accumulated epigenetic errors to induce transgenerational health issues [38]. The effects of nutrition and diet usually lead to notable transgenerational epigenetic errors in the insect models described above and in humans. Lumey and Stein first showed evidence of transgenerational inheritance of epigenetic errors in humans: grandchildren acquired epigenetic defects that had been transmitted from their grandmothers who had starved when pregnant because of war. The development of health disorders including obesity, glucose intolerance, and coronary heart disease in the grandchildren suggests intertwined relationships between nutrition and health status [39,40]. Similarly, a limited food supply has also been shown to induce transgenerational disorders that affect longevity of the children and grandchildren of the exposed mothers [41].

**Figure 1:**
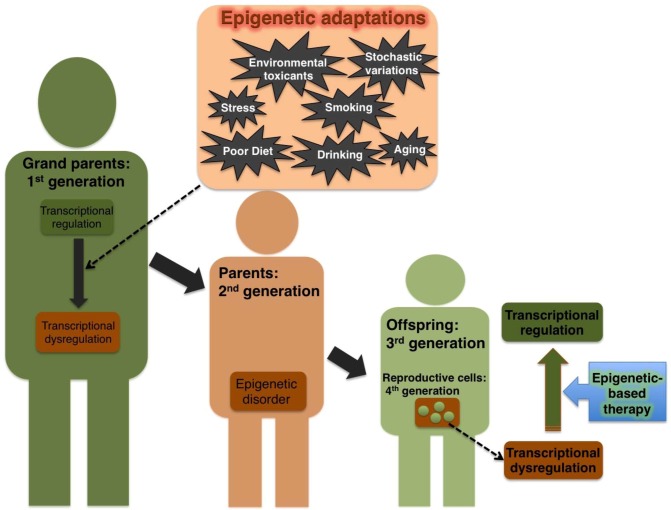
Transgenerational inheritance of epigenetic marks that leads to transcriptional dysregulation. Disadvantageous external stimuli (indicated in cream box) could cause aberrant regulation of the epigenome. The acquired epigenetic disorder (indicated in the red box) can be transmitted across generations from grand parents to their offspring. Epigenetic-based therapeutic approach may pave the way to restore this acquired transcriptional dysregulation even in the later generations (Restored transcriptional regulation is shown in green box).

Aging is the most prominent among other factors associated with epigenetic damage. During the aging process, when tissue damage occurs, stem cells tend to divide rapidly to replenish the impairment. The more the stem cells divide, the more prone they are to stochastic variation in their epigenetic pattern [42]. Most diseases of old people are likely to have an epigenetic or environmental component. Consistent with this notion, the aged humans with healthy habits are less prone to some common terminal illnesses such as cancer. Although lifestyle alone cannot restore health in older patients, their health can be improved considerably by changing lifestyle to include healthier habits. Therefore, the ability of behavior change to recover some disorders suggests potential clinical applications. A schematic representation of environment-mediated transgenerational epigenetic inheritance of transcriptional dysregulation and the epigenetic-based therapy to repair the disorders is shown in Figure 1.

## 3. Epigenetic-Switch Based Future Medicine

### 3.1. Co-Ordinated Histone Modifications

DNA methylation and/or histone modification along with the genomic imprinting are the key epigenetic changes that can switch the epigenetically inherited traits in an interdependent manner. In eukaryotic cell nuclei, DNA and histone proteins are packaged into nucleosomes, which form the chromatin structure. Histones encompass the core structural unit of chromatin and are classified into four major classes, histones H2A, H2B, H3 and H4, and linker units that are classified into histone H1/H5 [43]. The histone tails protrude from the nucleosome structure, and their modifying enzymes can influence gene expression *via* specific posttranslational modifications that includes methylation, acetylation, phosphorylation, citrullination, SUMOylation, ubiquitination and ADP-ribosylation [43]. The transcriptional state of chromatin is thought to be governed through the specific patterns of individual or combined histone modifications that occur at the right place and time to orchestrate the patterned gene expression *via* a hypothetical histone code [44].

**Figure 2:**
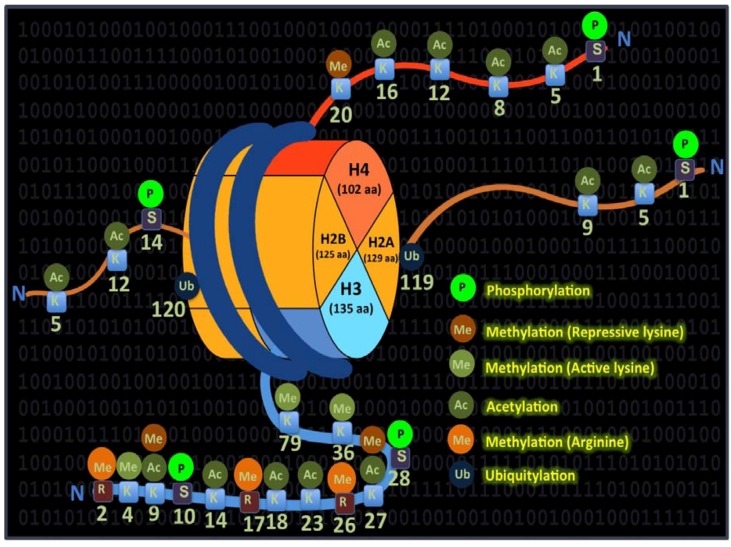
Colorful language of histone modifications and their precise coordination are suggested to constitute a code. Components of the core histones (H2A and B, H3 and H4) and their modifications are indicated in different colors. Together, these dynamic modifications are thought to comprise an imaginary code termed, the “histone code”, which is depicted as binary digits in the background.

Coordinated chromatin modifications can have different functional implications in different contexts [45]. Thus, the same chromatin area could be bivalent and harbor both transcriptionally permissive and nonpermissive histone modifications. In addition, specific histone modifications can recruit multienzyme complexes, which may affect other residues on the same or adjacent histone and this biologically significant phenomenon is known as “histone crosstalk”. Hence, assigning functional specificity to posttranslational modifications is not an easy task. Among these defined modifications, histone lysine acetylation is normally associated with a chromatin state that is permissive to transcription. Accordingly, the enzyme histone acetylase (HAT) is traditionally associated with gene activation as acetylation of lysine residues particularly in histone H3 and H4 increase the space between the nucleosome and the DNA that is wound around it. Histone deacetylase (HDAC) is associated with gene repression because it decreases the space between the nucleosome and the DNA. Acetylation of the core histone proteins is associated with both gene activity and biologically important functions such as chromatin assembly, DNA repair, recombination, and replication timing, all of which affect transcriptional competence [46,47,48]. HDAC-HAT equilibrium is essential in all the above-mentioned genome-related functions, and histone modifications have been suggested to constitute a code that governs the cellular phenotype. Individual histone modifications that are suggested to co-ordinate and confer the code are illustrated in Figure 2.

### 3.2. HDACs and Cancer

Cancer is the most studied of the diseases associated with miswriting of the histone code [49]. In the past, cancer was thought to be a disease associated with DNA damage that occurs through mutations of nucleotide sequences. In 1983, Feinberg *et al*. first demonstrated that an altered epigenomic pattern could distinguish a cancer cell from a normal cell [50]. Ever since this discovery, epigenetics has become an integral part of human cancer genetics. Histone acetylation can potentially shift the global balance of genome-wide gene expression from an oncogenic to an oncosuppressive state. Consequently, it is reasonable to assume that modulation of HDAC activity can induce dramatic changes in cellular phenotype. The essential role of HDAC in controlling the cell fate decision was demonstrated clearly by a study of acute paromyelocytic leukemia [51], which is caused by fusion proteins of retinoic acid receptor-α (RAR). Recruitment of HDAC can alter the retinoic acid (RA)-signalling pathway to induce myeloid differentiation [52]. Accordingly, pharmacological doses of RA were shown to decrease the turnover of fusion proteins by inducing the dissociation of HDAC from RAR [52]. The combined use of RA and HDAC inhibitor was suggested as a general strategy to treat myeloid leukemia. As mentioned previously it is not straightforward to generalize the functional relevance of histone modifications because HDAC inhibition might also negatively regulate gene expression. For example, in some cases like B-cell lymphomas, direct acetylation of p300 could hamper the oncogene BCL6 by disrupting HDAC activity [53].

HDAC activity can also be influenced by overexpression of HDAC-associated factors such as the gene of the metastasis-associated protein-1 (MTA-1), which causes changes in the mammary gland that result in tumor cells with a metastatic phenotype [54]. MTA-1 integrates the enzymatic activity of HDAC2 with the Wnt signalling pathway. Also, the role of HDAC2 in protecting cancer cells against apoptosis has been demonstrated in gene knockout studies [55]. In many tumors, upregulation of MTA-1 is associated with marked decreases in the levels of monoacetylated and trimethylated forms of histone H4. Some distinguishable histone modification patterns are also associated with the risk of prostate cancer recurrence in some patients [56]. In many forms of cancer, aberrant hypoacetylation of histones and nonhistone proteins, and HDAC recruitment to the promoter regions of certain tumor suppressor genes like p53 are often observed. In particular, mutations of HAT isoforms such as p300 decrease acetylation, leading to the formation of fusion proteins that promote carcinogenesis such as MLL-p300 and MOZ-p300, which are involved in aberrant gene expression. For example, in the normal state, MOZ controls the expression of RUNX1, which is involved in cellular differentiation, whereas the fusion protein MOZ-CBP induces the repression of this protein and thus promotes carcinogenesis [57].

Other epigenetic modifications such as methylation in DNA and histones can also occur simultaneously to silence tumor suppressor genes, which in turn can induce cell proliferation. In 2004, the FDA first approved the use of the DNA methyltransferase inhibitor “azacitidin”, which causes DNA methylation, to treat patients with myelodysplastic syndromes (MDS), a cancer that is associated with disordered hematopoiesis. Like most other cancers, MDS occurs in older people whose cells are prone to undergo rapid cell divisions and hence, frequent epigenetic adaptations [58]. Several HDAC inhibitors to be detailed in the subsequent section are effective in treating MDS. Because this cancer occurs in the blood-an accessible compartment-its treatment is relatively successful.

### 3.3. HDAC Inhibitors and Cancer Treatment

**Figure 3:**
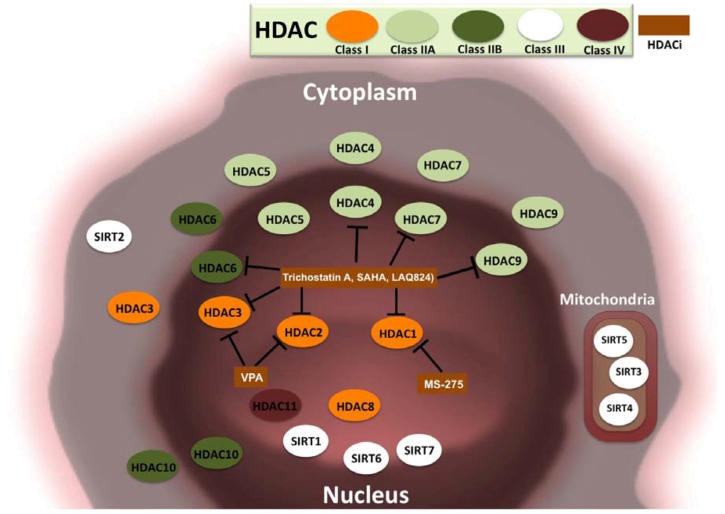
HDAC classes, their cellular localization, and inhibitory activities of certain HDAC inhibitors. A total of 18 HDACs belonging to four major classes of HDACs and their localization in cells are illustrated. HDACs that can localize to the cytoplasm and nucleus are indicated in both locations. Some notable HDAC inhibitors (HDACi) like trichostatin A, SAHA, NVP-LAQ824, valproic acid (VPA), MS-275 and their target HDACs on which they exhibit potent activity are also indicated.

HDAC can be broadly classified into four classes, depending on sequence identity and domain organization, as class I, IIA, IIB, III and IV [59]. Class I HDACs includes HDAC1, 2, 3 and 8. Class IIA HDACs includes HDAC4, 5, 7 and 9. Class IIB HDACs includes HDAC6 and 10. Class III HDACs include sirtuins (SIRT1, 2, 3, 4, 5, 6 and 7). Sirtuins possess HDAC or mono-ribosyl transferase activity and are implicated in the regulation of transcription, apoptosis and stress resistance. HDAC IV includes HDAC11. HDAC inhibitors are natural or synthetic small molecule that target one or more HDAC classes. HDAC inhibitors are used to modulate chromatin topology and global-level gene transcription Small molecules including hydroxamic acid (trichostatin A), cyclic tetrapeptides, benzamides, electrophilic ketones and the aliphatic acid compounds (valproic acid) classically act on class I and II HDACs. Suberoylanilide hydroxamic acid (SAHA) also called, vorinostat, is a second-generation hydroxamic acid derivative and was the first FDA-approved drug to treat cutaneous T-cell lymphoma (CTCL). SAHA is effective in the treatment of MDS and acute myelogenous leukemia when combined with the topoisomerase inhibitor idarubicin and cytarabine, a human deoxycytidine mimic that interferes with DNA to effectively kill cancer cells. Promising results were obtained recently in a phase I clinical trial of vorinostat in patients with advanced tumors even when used as the single chemical agent [60]. In phase II clinical trials, vorinostat was successful in treating metastatic breast carcinoma, glioblastoma and neck and head cancer [61]. Some of the most used HDAC inhibitors belonging to different class of compounds, the cancer type they act on and their clinical limitations are summarized in Table 1 [62,63,64,65,66,67,68]. The cellular localization of all classes and subclasses of HDACs and the target HDACs of some notable HDAC inhibitors are shown in Figure 3.

**Table 1:** Some selected HDAC inhibitors that have been used successfully in cancer treatment and side effect in patients exposed to these HDAC inhibitors.

Inhibitors and compound type	Cancer type	Clinical limitations	References
Short chain fatty acids			
Sodium butyrate	Leukemia, myeloma and breast cancer	Combination therapy is toxic	[62]
Sodium Phenyl butyrate	Leukemias and myelodysplasia	High doses causes neurological toxicity	[63]
Sodium valproate	Leukemias, myelo dysplasia and cervical cancer	Neurological toxicity	[64]
Hydroxamic acids			
Suberoylanilide hydroxamic acid	Leukemia, lymphoma and solid tumors	Dose limiting toxicity, dehydration, fatigue, diarrhoea and anorexia.	[65]
NVP-LAQ824	Leukemia, lymphoma and solid tumors	Toxicity in bone marrow and cardiac cells. Fever, fatigue and nausea	[66]
PXD101	Advanced solid tumors	Tiredness, fatigue and low-grade nausea	[67]
Others			
MS-275	Leukemia	Toxicity, nausea and vomiting	[68]

### 3.4. Epigenetic Switches in Cellular Reprograming

Parallels have long been drawn between cancer cells and stem cells as they display superficial similarities. Both cancer and stem cells are capable of self-renewal and can migrate inside the body. Stem cells can switch to become cancer cells under certain microenvironments [69]. Among the diversity within the animal kingdom, the most remarkable is the capability of several vertebrates such as the fish, frog and salamander to regenerate complex body parts [70]. In general, mammals do not inherently possess this regenerative capacity. However, the rapidly advancing and epoch-making strategy of inducing pluripotency in human somatic cells suggests the possibility of numerous applications in regenerative medicine [71,72]. Regeneration involves dynamic cellular and transcriptional reprogramming processes that reset the heritable epigenetic maintenance of genome-wide gene expression [73]. Many factors that could perturb and reset the epigenetic modifications have been shown to enhance the generation of iPSCs [74]. In particular, small molecules that can inhibit enzymes such as HDACs markedly increase the efficiency of iPSC generation [75,76]. The HDAC inhibitors used to improve somatic cell reprogramming efficiency and differentiation are summarized in Table 2 [77,78,79,80,81,82,83,84].

**Table 2:** HDAC inhibitors and their activity and uses in regenerative medicine.

Inhibitors	HDAC activity	Uses	Comments	References
**Aliphatic chain fatty acids**				
Sodium butyrate (NaB)	Inhibit most HDACs except class III and HDACs 6 and 10 of class II	Cellular reprogramming, differentiation and self renewal	Dramatically enhances reprogramming efficiency in both mouse and human embryonic stem cells	[76,77]
Valproic acid (VPA)	Both Class I and II with higher potency against HDACs 2 and 3	Cellular reprogramming, differentiation and self renewal	Self renewal of embryonic carcinoma cells, enhance reprogramming efficiency with fewer factors and induce neuronal differentiation	[75,78]
**Hydroxamic acids**				
Trichostatin A	Higher potency against HDACs 1,2,3,4, 6,7 and 9 and active against HDAC8.	Cellular reprogramming, differentiation and self renewal	Self renewal of mouse embryonic stem cells, embryonic carcinoma cells and neurosphere cells	[79,80,81]
Suberoylanilide hydroxamic acid	Higher potency against HDACs 1,2,3,4, 6,7 and 9 and active against HDAC8.	Cellular reprogramming and differentiation	Enhances reprogramming efficiency and Induces neuronal differentiation	[82]
Scriptaid	Class I and II with higher potency against HDACs 1, 3, 6 and 8.	Cellular reprogramming	Enhances reprogramming efficiency	[81]
Oxamflatin	Class I and II with higher potency against HDACs 1, 3, 6, 7 and 8.	Cellular reprogramming	Enhances somatic nucleus reprogramming	[80]
M344	Class I and II with higher potency against HDACs 1,2 and 3	Cellular differentiation	Induces neuronal differentiation.	[82]
M-Carboxycinnamic acid bishydroxamide (CBHA)	Class I and II with higher potency against HDACs 1 and 3	Cellular reprogramming	Enhances reprogramming efficiency	[84]
**Others**				
Apicidin	Higher potency against HDACs 2 and 3	Cellular reprogramming	Enhances reprogramming efficiency	[75]
Chlamydocin	Higher potency against Class I	Self renewal	Self renewal of hematopoietic stem cells	[83]
MS-275	Higher potency against class I especially HDAC1	Cellular reprogramming and differentiation	Induces neural differentiation	[75,82]

Increasing evidence suggests that major regions of the iPSC epigenome do not revert to the embryonic state but instead retain the epigenetic memory of their tissue of origin [85]. A recent study suggests that chromatin-modifying enzymes can act as both facilitators and barriers to epigenetic remodeling of differentiated cells to a stem cell configuration [86]. Epigenetic reprogramming is believed to be the key to improve the clinical utility of iPS cells as they specifically control the transcriptional network that confers to pluripotency. Hence, epigenetic-based drugs that could precisely modulate the complex transcriptional network in a sequence-specific manner could efficiently reprogram cells from fibroblasts to a pluripotent state [87]. One such prospective strategy to use sequence-specific small molecules with epigenetic activity is already under development and is discussed later in this review.

## 4. Tailor-Made Epigenetic-Based Switches

### 4.1. Need for Selectivity

Notwithstanding the excitement surrounding the use of histone modifying enzyme inhibitors in reverting epigenetic adaptations, lack of selectivity is a major concern. HDAC inhibitors are relatively selective to cancer cells; that is, these drugs do not act on proteins to acetylate all sorts of genes and create chaos in the body [88]. Therefore, the effectiveness of HDAC inhibitors can differ because they have a greater effect on highly responsive cancer cells than the relatively less responsive normal cells. In this regard, care should be taken before considering epigenetic-based therapeutic approaches in young children and pregnant woman because of the potential serious side effects. Accessibility of drugs is another bottleneck that hampers the treatment of some cancers such as breast cancer; hence, epigenetic-based drugs should be designed to target the genes involved efficiently. Some genetically induced cancers require drugs that are precise to specific genes, whereas cancers associated with epigenetic modifications require different drugs [89]. In this context, HDAC inhibitors that are selective for specific DNA sequences could be developed for effective therapeutic gene modulation.

### 4.2. Programmable Genetic Switches To Reset Epigenome

Our laboratory has been developing selective DNA-binding hairpin pyrrole-imidazole polyamides (PIPs) as artificial genetic switches to precisely upregulate gene(s) of interest. PIPs bind to the minor groove of DNA according to the rule that an antiparallel pairing of I opposite P (I/P) recognizes a G–C base pair, and a P/P pair recognizes A–T or T–A base pairs. PIPs have binding constants similar to those of natural transcription factors and can permeate living cells where they can modulate endogenous gene expression [90]. Consequently, PIPs can be used to regulate gene expression in a sequence-specific manner. Synthetic PIPs possessing an alkylating moiety have been demonstrated to induce sequence-specific gene silencing in mammalian cells [87,90]. Anticancer activities of alkylating PIPs that were designed to target various cancer-associated genes were also demonstrated [91].

**Figure 4:**
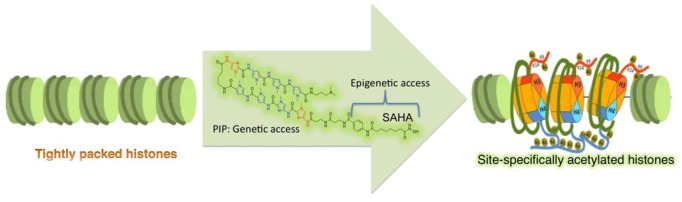
Site-specific histone modifications using SAHA-PIP. SAHA-PIP encompasses chemical moieties that could access both epigenetic and genetic environments, and can be tuned to specific DNA sequences for inducing site-specific histone modifications.

In nature, gene regulation is achieved at various distinctive levels, and programmable gene-based transcriptional activators have been overlooking the critical epigenetic influence in gene control [87]. Using a novel strategy, we tried to mimic the natural cellular environment and to devise a strategy to increase the selectivity of the HDAC inhibitor SAHA by conjugating it with sequence-specific PIPs to generate a new class of compounds, termed SAHA-PIPs. We evaluated the biological activity of SAHA-PIP, which was designed to target the promoter region of the tumor suppressor gene p16 in HeLa cells was evaluated. The SAHA-PIP designed to match the promoter region induced sequence-specific acetylation in the promoter region of p16 and caused a significant morphological change in HeLa cells [92]. Mismatched SAHA-PIP and SAHA alone did not induce such activity to suggest the sequence-specific capability of SAHA-PIP (Figure 4)

Encouraged with these results, a library of 16 SAHA-PIP was synthesized. Since the enforced expression of pluripotent factors has been shown to switch the cell fate, we chose *Oct-3/4*, *Nanog*, *Sox2*, *Klf4* and *c-Myc* as the target genes to evaluate the biological activity of the designed SAHA-PIPs in mouse embryonic fibroblasts (MEFs). Certain SAHA-PIPs differentially induced pluripotent gene expression through the initiation of epigenetic marks that confers to transcriptionally permissive chromatin including histone H3 Lys9, Lys14 acetylation and Lys4 trimethylation [93]. Subsequently, screening studies were performed by altering the chemical structure of the hit SAHA-PIP, and the result suggested that chemical modifications in SAHA-PIP could improve the expression level of the target pluripotent genes [94]. The scope of improvement demonstrated in this work confirmed the possibility of tailoring programmable SAHA-PIPs to improve their efficacy.

Screening of a second library of SAHA-PIPs with improved recognition of GC-rich sequences led to the identification of a potent SAHA-PIP that could rapidly induce multiple pluripotency genes. Genome-wide gene analysis revealed that the hit SAHA-PIP, termed δ, shifted the transcriptional network from the fibroblast to the dedifferentiated state in just 24 h. Surprisingly, δ-treated MEFs rapidly overcame the mesenchymal epithelial transition (MET) stage, an important rate-limiting step during dedifferentiation of the somatic genome [95]. It is important to note here that MET is not only associated with iPS cell reprogramming but also are known as the potential therapeutic target in the prevention of metastases.

Epithelial cells are more flexible for reprogramming because they have already acquired some of the characteristics of pluripotent cells [96]. Hence, strategies to expand our SAHA-PIP(s) may lead to effective switching of cellular state to the differentiated or proliferative state with the need of fewer factors. A recent report also indicated that cells differentiated from cells with pre-existing features of pluripotent stem cells displayed superior and more rapid gliogenic competency compared with those differentiated from either iPSCs or directly from somatic cells [97]. Hence, SAHA-PIPs might induce and/or improve the reprogramming efficiency to attain pluripotent stem cells with all the power of embryonic stem cell but without the need for embryos.

## 5. Future Strategies To Tinker with Epigenetic Inheritance

### 5.1. Heritable Histone Modifications as Effective Therapeutic Targets

The epigenome of embryonic stem cells and fibroblasts have been studied extensively. However, there are hundreds of cell types, and it is possible that each cell type could have a different epigenome [98]. Ongoing basic and clinical research has suggested the importance of epigenetic inheritance and the need to devise novel therapeutic strategies to modulate this inheritance [99]. Studies to explore the mechanisms behind the establishment and maintenance of epigenetic marks are progressing at astonishing pace to achieve durable epigenetic reprogramming of the acquired transgenerational epigenetic marks.

Therapeutic targeting of epigenetic inheritance has several advantages over classical inheritance because these induced adaptations can be transmitted across generations [13], giving offspring the ability to meet the environmental challenges that affected their parents. However, the epigenetically induced signalling pathway and/or phenotype should match the environmental changes. For example, epigenetic inheritance of elevated insulin sensitivity acquired by mothers in the food-scarce environment provides protection for the offspring under a similar scarce environment [100]. On the other hand, development of this phenotype that does not match the predicted environment could lead to obesity and diabetes. Consistent with this concept, epigenetic alterations acquired by the offspring as a result of stressful lifestyle in the parent(s) could be dangerous if the offspring do not encounter a similar stressful environment. Nevertheless, epigenetic alterations associated with lifestyle, such as excess consumption of alcohol or exposure to environmental toxicants, could lead to undesirable and durable transgenerational effects that are difficult to restore and reset [100,101].

The concept of epigenetics bridges the gap between nature and nurture, and helps us understand disease risk better [102]. Although, the rise of epigenetics further complicates an already complex system, understanding epigenetic mechanisms could lead to effective disease diagnostics and early prediction of disease susceptibility. For instance, detection of differential methylation pattern in the brains of a patient who is a victim of child abuse could help us to diagnose and prevent their suicidal behavior [103].

Earlier diagnosis of the transgenerational epigenetic alterations could lead to effective manipulation of epigenetic marks that are easier to revise than the gene mutations or single nucleotide polymorphisms. For cancer treatment, several epigenetic drugs have already been approved [104], although most are broad and nonspecific, and have undesirable side effects [105]. In the case of complex disorders associated with depression, some drugs including HDAC inhibitors, are already accepted for treatment [106]. Psychiatric diseases are strongly associated with the epigenetic alterations, and hence, devising new strategies are warranted [107].

Although several examples that support the existence of the transgenerational inheritance of epigenetic marks have been demonstrated in animal models, their actual existence is yet to be clarified in humans. Epigenetic marks have been shown to be linked to the DNA sequence of the genome and to probabilistic and environmental events. Recent evidence suggests that transgenerational epigenetic inheritance might also occur through gametes. The development of modern technologies such as high-throughput sequencing opens up the possibility of tracing the molecular nature of the epigenetic marks. Some compelling evidence suggests that these epigenetic marks could occur through diffusible factors, in particular RNA. Also, transgenerational epigenetic inheritance via gametes has been demonstrated to occur mainly at retrotransposons and other repeated elements. Hence, strategies that consider both the epigenome and the genome may allow us to develop better tools for predicting phenotype at an individual level [108].

### 5.2. Dynamic Histone Modifications – Can It Be Therapeutically Targeted?

Modifications induced by histone-associated enzymes are prone to environmental influence and histones can change at any time [49]. Hence, therapeutic targeting of histone modulating enzymes is complicated, as a single gene could undergo hundreds of epigenetic modifications. Currently, epigenetic-based therapy is considered the future of medicine because of the possibility of restoring the epigenetic errors of the patients when combined with gene therapy. Completely erasing the errors and thus returning the cell to the normal state could avoid inheritance of this accumulated epigenetic burden by the next generation. The major advantage of epigenetic-based therapy is that, unlike the painful chemotherapeutic approach, no killing of cells is involved. The search is now on to decode the more complex pattern of modifications that accumulate progressively over time, which dictate the global changes in the expression of genes associated with cancer. Understanding the elusive epigenetic code could help us to understand the mechanism underlying the initiation of cancer. Accessibility of drugs is another major issue for some types of cancer such as breast cancer; future design of epigenetic drugs should also focus on accessibility. In this regard, histone deacetylase inhibitors that are selective to specific DNA sequences and cell permeable like the SAHA-PIPs that were mentioned before could be efficiently combined in the strategies to treat cancer.

### 5.3. Prospects of Tailor-Made Epigenetic Switches

Encouraged by the exciting results obtained with SAHA-PIP(s), it is reasonable to assume that the epigenome could be altered in a site-specific manner. Unlike other artificial transcription factors that fail to access their designated target sites due to repressive DNA methylation marks, PIPs have the ability to bind with packed chromatin architecture [109]. Since SAHA-PIPs encompass both sequence-specificity and epigenetic activity, they could be used to shift the transcriptional network from one cell state to another in a precise manner. Since chromatin modifications govern the specification of cell fate, site-specific chromatin modifications might be able to modulate the cellular phenotype, for example, the sequence-specific induction of pluripotent stem cells. The problem of epigenetic memory, which hampers the clinical translation of iPSCs, could potentially be resolved by epigenetic reprogramming in a sequence selective manner using SAHA-PIPs.

**Figure 5:**
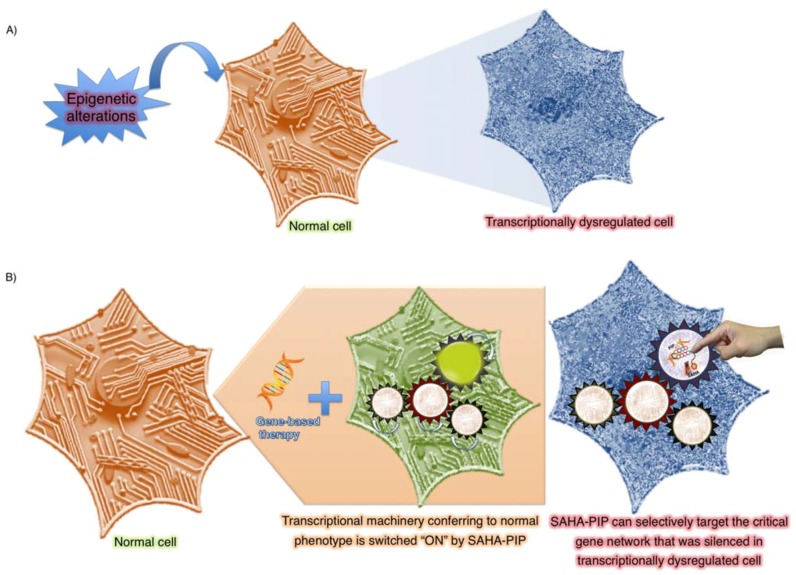
Proposed strategy to modulate transgenerational epigenetic inheritance A) Epigenetic alterations could switch the cellular transcriptional machinery (Illustrated as diodes) from the normal (Orange) to the dysregulated (Blue) state. B) SAHA-PIP that was previously shown to induce multiple genes pertaining to single network [95] could potentially activate the pre-silenced gene network indicated as coordinated machine wheel that is critical for resetting the transcriptional dysregulation. This epigenetic-switch based approach alone may be insufficient to revert back the diseased cell to the normal state but when combined with gene-based approach, it may serve as the sustainable solution to some unresolved clinical issues.

PIPs could also be tailored with ease using automation-driven solid phase synthesis. Further tuning is also possible because they possess flexible sites for covalent attachment to other molecules. It is also possible to attach other histone- and DNA-associated enzymes and signalling inhibitors, which could be driven to the specific DNA sequences that form the core of a specific transcriptional network that controls cellular phenotype. For example DNA methyl transferase inhibitors such as 5-azacytidine, RG-108 and other small molecule inhibitor could be conjugated with PIPs. These chromatin-modifying PIPs also called, as CM-PIPs, may be able to epigenetically silence the selective gene(s) that are overexpressed in certain disorders such as cancer [87]. However, correcting abnormal chromatin modifications alone may be insufficient to allow the cell to revert back to the normal state. Hence, a combined approach that encompasses the correction of errors in both the genome and epigenome could lead to the development of sustainable strategies to treat cancer effectively (Figure 5). Nevertheless, some bottlenecks including the cell permeability of PIPs [110] and genome-wide specificity should be considered before the use of tailor-made PIPs as effective genetic switches.

## 6. Conclusions

The importance of a dynamic epigenome and its role in modulating cell destiny has been realized recently. Complex interactions between the genetic make-up and the environment may lead to the inheritance of certain epigenetic marks through the germline, which may manifest during early development or later in life [111]. Epigenetic errors play a key role in a wide variety of diseases and several inflammatory and metabolic disorders. Epigenetic alterations attributed to the modern lifestyle are not always harmful, and some beneficial alterations such as restoration of epigenetic health by repression of signalling pathways conferring to inflammation are also known [89]. Clinicians are hopeful because future strategies suggest that by toggling the biochemical switches it may be possible to erase epigenetically inherited disorders in dysregulated cells to give the next generation a new start. Recent compelling evidence disfavors the Darwinian principles that advocate that the information transcribed from the genetic code cannot be modified. It is possible that epigenetic inheritance mechanisms are a specialized form of phenotypic plasticity that cannot pioneer evolutionary novelty into a species lineage [112]. Nevertheless, gaining insights into the epigenetic switching of cell fate may help us develop personalized therapeutic strategies to restore the diseased cell to a normal state. One such fundamental area of interest is identifying how well the human body is engineered to establish tissue-specific epigenetic marks according to environmental experiences. Nature has orchestrated organized adaptive responses of evolution and has resolved the biological complications that were initially considered to be unfixable. Gaining insights about how signalling pathways are methodically coordinated in diverse organisms including humans could be useful for devising strategies to effectively manipulate human physiological pathways [113]. Many lessons are still to be learned to allow us to mimic from nature and to devise and achieve sustainable strategies for effective clinical translation of iPSC technology, and the treatment of cancer and several neurological diseases.
